# Everolimus downregulates estrogen receptor and induces autophagy in aromatase inhibitor-resistant breast cancer cells

**DOI:** 10.1186/s12885-016-2490-z

**Published:** 2016-07-16

**Authors:** Asona Lui, Jacob New, Joshua Ogony, Sufi Thomas, Joan Lewis-Wambi

**Affiliations:** Department of Molecular and Integrative Physiology, University of Kansas Medical Center, Kansas City, KS 66160 USA; Department of Anatomy and Cell Biology, University of Kansas Medical Center, Kansas City, KS 66160 USA; Department of Cancer Biology, University of Kansas Medical Center, Kansas City, KS 66160 USA; Department of Otolaryngology, University of Kansas Medical Center, Kansas City, KS 66160 USA; The University of Kansas Cancer Center, Kansas City, KS 66160 USA

**Keywords:** Breast cancer, Aromatase inhibitor, RAD001, Everolimus, PI3K/Akt/mTOR, Estrogen receptor, Autophagy

## Abstract

**Background:**

mTOR inhibition of aromatase inhibitor (AI)-resistant breast cancer is currently under evaluation in the clinic. Everolimus/RAD001 (Afinitor®) has had limited efficacy as a solo agent but is projected to become part of combination therapy for AI-resistant breast cancer. This study was conducted to investigate the anti-proliferative and resistance mechanisms of everolimus in AI-resistant breast cancer cells.

**Methods:**

In this study we utilized two AI-resistant breast cancer cell lines, MCF-7:5C and MCF-7:2A, which were clonally derived from estrogen receptor positive (ER+) MCF-7 breast cancer cells following long-term estrogen deprivation. Cell viability assay, colony formation assay, cell cycle analysis and soft agar anchorage-independent growth assay were used to determine the efficacy of everolimus in inhibiting the proliferation and tumor forming potential of MCF-7, MCF-7:5C, MCF-7:2A and MCF10A cells. Confocal microscopy and transmission electron microscopy were used to evaluate LC3-II production and autophagosome formation, while ERE-luciferase reporter, Western blot, and RT-PCR analyses were used to assess ER expression and transcriptional activity.

**Results:**

Everolimus inhibited the proliferation of MCF-7:5C and MCF-7:2A cells with relatively equal efficiency to parental MCF-7 breast cancer cells. The inhibitory effect of everolimus was due to G1 arrest as a result of downregulation of cyclin D1 and p21. Everolimus also dramatically reduced estrogen receptor (ER) expression (mRNA and protein) and transcriptional activity in addition to the ER chaperone, heat shock protein 90 protein (HSP90). Everolimus restored 4-hydroxy-tamoxifen (4OHT) sensitivity in MCF-7:5C cells and enhanced 4OHT sensitivity in MCF-7 and MCF-7:2A cells. Notably, we found that autophagy is one method of everolimus insensitivity in MCF-7 breast cancer cell lines.

**Conclusion:**

This study provides additional insight into the mechanism(s) of action of everolimus that can be used to enhance the utility of mTOR inhibitors as part of combination therapy for AI-resistant breast cancer.

**Electronic supplementary material:**

The online version of this article (doi:10.1186/s12885-016-2490-z) contains supplementary material, which is available to authorized users.

## Background

Estrogen deprivation using aromatase inhibitors (AIs) is currently the standard of care for patients with estrogen receptor-positive (ER+) breast cancer. Unfortunately, ~30 % of breast cancer patients develop resistance to AIs following long-term treatment [1]. The mechanism by which AI resistance develops is still not completely understood, however, several contributing factors have been identified including; alterations in ER signaling, enhanced growth factor signaling, and imbalance in the phosphoinositide 3-kinase/protein kinase B/mammalian target of rapamycin (PI3K/Akt/mTOR) pathway [2, 3]. The activation of the PI3K/Akt/mTOR pathway is considered clinically relevant for tumor escape from hormone dependence in breast cancer, promoting the survival of breast cancer cells in estrogen-deprived conditions [4]. Additionally, upregulation of the PI3K/Akt/mTOR pathway is associated with poor outcome for breast cancer patients and has been observed in AI-resistant breast cancer models [5, 6]. As a result, a variety of PI3K/Akt/mTOR pathway inhibitors have been under study, including everolimus/RAD001 (Afinitor®).

Everolimus is a rapamycin analog that is currently approved for treatment of metastatic breast cancer. It inhibits the PI3K/Akt/mTOR signaling pathway by preventing the phosphorylation of mTORC1, which interrupts the signaling cascade and results in inhibition of cell proliferation and growth [7]. Everolimus treatment has shown promising anti-cancer effects in preclinical studies; however, when used as a single agent, it does not significantly decrease tumor size [8]. As a result, recent clinical trials have focused instead on simultaneous targeting of the PI3K/AKT/mTOR and ER pathways in ER+ breast cancer [9–11]. The results from these trials have been very encouraging due to significant improvements in response rate and progression free survival for both AI-sensitive and AI-resistant patients [12–14]. Subsequent laboratory studies have focused on comparison of everolimus in combination with endocrine therapies [15, 16] as well as other PI3K/Akt/mTOR inhibitors in a variety of breast cancer cell lines [17, 18] and these studies have reported synergy between tamoxifen or AI therapy and everolimus. However, these studies have not investigated the anti-cancer mechanisms of everolimus alone in AI-resistant breast cancer cells.

Everolimus and other PI3K/Akt/mTOR inhibitors are known to induce autophagy in both solid and blood tumors [19, 20]; however, to our knowledge, the phenomenon has not been reported in breast cancer. Autophagy allows cells to degrade dysfunctional organelles and proteins, and recycle their components. Autophagy can support tumor survival during treatment, making it a possible mechanism for AI-resistance [21, 22]. This process is dependent upon the cleavage of microtubule associated light chain 3 (LC3) to LC3-I and subsequent lipidation to LC3-II which allows for final formation of the autophagosome membrane [23]. A group of small proteins, called heat shock proteins (HSPs), promote cell survival during stress reactions by promoting the refolding of denatured proteins and directly regulating autophagy. Specifically, HSP70 is thought to be required for the induction of autophagy [24, 25] in response to inhibition of the PI3K/Akt/mTOR pathway by either starvation or rapamycin treatment [26, 27]. Another heat shock protein, HSP27, allows cells to survive a variety of cytotoxic stimuli [28, 29] and is thought to be degraded during starvation and rapamycin-induced autophagy [30]. Due to the link between drug resistance and autophagy, we hypothesized that the induction of autophagy may contribute to everolimus insensitivity in MCF-7 breast cancer cell lines.

In this study, we investigated the effects of everolimus, as a single agent, or in combination with 4-hydroxy tamoxifen (4-OHT) or chloroquine on cell proliferation, anchorage-independent growth, PI3K/Akt/mTOR signaling, ER expression and transcriptional activity, LC3 turnover, and autophagosome induction in AI-sensitive MCF-7 and AI-resistant MCF-7:5C and MCF-7:2A breast cancer cells. We report that everolimus exerts similar anti-proliferative effects in both the AI-sensitive and AI-resistant breast cancer cell lines and that its inhibitory activity is associated with G1 arrest and down regulation of ERα expression. Everolimus also reverses and enhances 4OHT sensitivity during long-term co-treatment of the AI-resistant cell lines. Lastly, we report that autophagy is a mechanism of everolimus insensitivity in MCF-7, MCF-7:5C and MCF-7:2A cells, possibly explaining the equal response of these cell lines to treatment. The information from this study may enhance future selection of everolimus containing combination therapies for AI-resistant breast cancer.

## Methods

### Cell lines and culture conditions

The MCF-7 cell line [31, 32] was obtained from Dr. V. Craig Jordan (University of Texas MD Anderson Cancer Center, Houston) and maintained in RPMI-1640 medium supplemented with 10 % fetal bovine serum, 2 mM glutamine, Antibiotic/Antimitotic mix, MEM Non-Essential Amino Acids (Invitrogen, Waltham, MA), and bovine insulin at 6 ng/mL (Sigma Aldrich, St. Louis, MO). The long-term estrogen deprived human breast cancer cell lines; MCF-7:5C [31, 33] and MCF-7:2A [32, 34] were cloned from parental MCF-7 cells following long term (>12 months) culture in estrogen-free medium composed of phenol red-free RPMI-1640, 10 % fetal bovine serum treated three times with dextran-coated charcoal (SFS), 2 mM glutamine, bovine insulin at 6 ng/mL, Antibiotic/Antimitotic mix, and MEM Non-Essential Amino Acids (Invitrogen). The MCF10A cell line was purchased from the American Type Tissue Culture Collection. They are maintained in Dulbecco's Modified Eagle Medium: Nutrient Mixture F-12 (DMEM/F12) in a 1:1 mixture and supplemented with 5 % horse serum, Antibiotic/Antimitotic mix (100 IU/mL penicillin, 100 μg/mL streptomycin, 25 μg/mL of Fungizone® from Invitrogen, Grand Island, NY), 20 ng/ml EGF (Millipore), 0.5 mg/ml hydrocortisone, 100 ng/ml cholera toxin (Sigma Aldrich). All cell lines were cultured at 37 °C under 5 % CO_2_. After overnight acclimatization period, cells were cultured with 20 nM everolimus alone or in combination with 1 μM 4-hydroxytamoxifen (Sigma Aldrich) or 50 μM Chloroquine (InvivoGen, San Diego, CA), in their normal culture medium.

### Cell viability

Cells were assayed for viability in 24-well plates using the Cell-Titer Blue Assay Kit (Promega, Madison, WI) per the manufacturer’s instructions. Assay plates were kept at 37 °C in 5 % CO_2_ for 3 h and read at 560–590 nM on a BioTek Synergy 4 microplate reader using the Gen 5 data analysis software (BioTek Instruments, Winooski, VT).

### Western blotting

Cells were seeded in 6-well plates, collected using a cell scraper and suspended in RIPA buffer (Thermo Scientific, Pittsburgh, PA) supplemented with protease inhibitor cocktail and phosphatase inhibitor (Sigma Aldrich). Cells were homogenized over ice by sonication. After purification of the sample by centrifugation, protein concentration was determined by protein assay (Bio-Rad, Hercules, CA). The proteins were separated by 4-12 % SDS–polyacrylamide gel electrophoresis (SDS–PAGE) and electrically transferred to a polyvinylidene difluoride membrane (Santa Cruz Biotechnology). After blocking the membrane using 5 % non-fat milk, target proteins were detected using either Anti-mTOR, anti-phospho-mTOR, anti- LC3A/B (Cell Signaling, Beverly, MA), anti-p70S6K, anti-phospho-p70S6K, anti-AKT, anti-phospho-AKT (S473) or anti-ERα (Santa Cruz Biotechnology) antibodies. Membranes were stripped and re-probed for β-actin (Cell Signaling) or β-tubulin (Sigma Aldrich). The appropriate horseradish peroxidase (HRP)-conjugated secondary antibody was applied and the positive bands were detected using Amersham ECL Plus Western blotting detection reagents (GE Health care, Piscataway, NJ). In the case of LC3 analysis, cells were treated with 50 μM chloroquine (CQ) for 24 or 48 h to allow for LC3-II accumulation. Immunoreactivity was detected using anti-mouse or anti-rabbit IgG conjugated to Dylight 680 or 800 fluorochromes (Thermo Scientific, Waltham, MA), respectively. Blots were visualized on Odyssey imager (LiCor, Lincoln, NE). Quantitation of immunoreactive signals was done by densitometry using ImageJ 1.46r software (NIH, Bethesda, MA). The ratio of protein expression to β-tubulin in each lane was calculated and presented relative to the respective controls within each experiment.

### Cell cycle analysis

Cells were incubated in the appropriate cell culture media with and without drug treatment. Cells were harvested at the indicated time points by trypsinization. They were washed once with cold PBS and stained with 50 μg/mL Propidium Iodide and 100 μg/mL RNase A in PBS (Invitrogen). Samples were analyzed using a BD FACSAria™ II Flow Cytometer (BD, Franklin Lakes, NJ) and the data were analyzed with FlowJo software (Ashland, OR).

### Clonogenic proliferation assay

Cells were seeded at 1,000 cells per well in 6-well plate in singe cell suspension. After 24 h acclimatization, they were treated every three days and allowed to proliferate and establish colonies for 9 days. Cells were stained with 0.5 % crystal violet in 1:7 acetic acid: methanol and imaged at 1X in a Bio-Rad ChemiDoc™ XRS+ System with Image Lab™ Software (Bio-Rad Laboratories Inc., Hercules, CA). Colonies were counted and measured using Image J software (The National Institute of Health, Bethesda, MD).

### Soft agar anchorage-independent growth assay

6-well plates were coated with 1 mL of 0.8 % agarose in the appropriate culture media. Cells were then suspended in 0.48 % agarose and immediately overlaid on the pre-coated plates. Once the agarose was solid, 1 mL of culture medium with or without 20 nM everolimus was added and replaced every 4 days for 15 days. Cultures were then stained with 0.005 % crystal violet in PBS, washed with PBS until the background was clear and imaged microscopically at 10X for measurement of colony diameter. Plates were also imaged at 1X in a Bio-Rad ChemiDoc™ XRS+ System with Image Lab™ Software (Bio-Rad). Colonies were counted and measured using Image J software (NIH).

### Real time PCR

Cells were seeded in 6-well plates and allowed to acclimatize overnight. Following 72 h treatment with 20 nM everolimus, cells were harvested by cell scraping in RLT lysis buffer and total RNA was isolated using the Qiagen RNeasy kit (Venlo, Limburg). First strand cDNA synthesis was performed from 3 μg total RNA using MulV Reverse Transcriptase (Applied Biosystems, Carlsbad, CA) on a Bio Rad MyCycler™. RT-PCR was conducted using the ViiA™ 7 Real-Time PCR system (Applied Biosystems) and SYBR Green Reagent (Life Technologies, Carlsbad, CA) with primers specific for ERα and the housekeeping gene PUM1. Primers for ERα: Forward 5’–AAGAGGGTGCCAGGCTTTGT–3’, Reverse 5’–CAGGATCTCTAG CCAGGCACAT –3’. Primers for PUM1: Forward-TCACCGAGGCCCCTCTGAACCCTA Reverse- GGCAGTAATCTCCTTCTGCATCCT (Integrated DNA Technologies, Coralville, Iowa). Relative ERα mRNA expression level was determined as the ratio of the signal intensity to that of PUM1 using the formula: 2^-ΔCT^. When treated with everolimus, fold change in ERα expression was normalized to PUM1 and then compared to the untreated value for that cell line using the formula: 2^-ΔΔCT^.

### Luciferase reporter assay

Cells were seeded in 12-well tissue culture plates overnight for attachment before transfection. The cells were transfected using Lipofectamine 2000™ Transfection Reagent (Invitrogen, San Diego) according to the manufacturer's recommendations. Briefly, 4 μL of Lipofectamine 2000, 0.8 μg of ERE Luciferase plasmid DNA and 0.01 μg of the pRL CMV Renilla (Promega) were diluted individually in 250-μl aliquots of OptiMEM Reduced-Serum Medium (Invitrogen). Cells were incubated for 24 h after transfection, and then treated with 20 nM EVE or vehicle in complete media for 24 h. The Luciferase and Renilla activities were measured using the dual luciferase assay kit (Promega) according to the manufacturer's instructions. To confirm the specificity of the ERE Luciferase construct, EVE treated cells were also compared to those treated with 1nM 17β-estradiol and 1nM Fulvestrant, a pure anti-estrogen (Sigma). Relative Fluorescence Units (RFUs) were calculated as a ratio of Luciferase to Renilla signal intensity. The ERE Luciferase reporter construct was a kind gift from Dr. Clodia Osipo (Loyola University, Chicago, IL).

### Immunofluorescence microscopy

Cells grown on glass coverslips were washed in PBS and fixed with 100 % ice cold methanol for 10 min. After permeabilization by 0.1 % Triton X-100 in PBS for 10 min, cells were incubated with 5 % normal horse serum/PBS for 30 min, followed by incubation with ERα or LC3B antibody, 2 μg/mL in 0.01 % Triton X-100 /PBS overnight (Santa Cruz Biotechnology). Cells were stained with fluorescein isothiocyanate (FITC)-conjugated labeled goat anti-rabbit IgG (Cell Signaling), 4 μg/mL in PBS for 1 h, followed by coverslip mounting with the ProLong® Gold anti-fade reagent with DAPI (Life Technologies). Samples were imaged on a Leica TCS SPE confocal microscope in the Confocal Imaging Core at The University of Kansas Medical Center. Images were collected and analyzed using the Leica LAS AF Lite software (Leica Biosystems, Nussloch, Germany). For quantification, mean fluorescent intensity was determined using Image J software on green (FITC) channel images.

### Electron microscopy

1 x10^6^ cells were seeded in 6-well plates and treated after an overnight acclimatization. After 72 h of treatment cells were harvested by scraping and fixed for 24 h at 4 °C in 0.1 M cacodylate buffer supplemented with 2 % glutaraldehyde. Samples were then processed in The Electron Microscopy Research Laboratory at KU Medical Center as follows. Briefly, cell pellets were washed in 0.1 M cacodylate buffer for 10 min, and resuspended. Cell pellets were post fixed in 1 % osmium tetroxide buffered in 0.1 M cacodylate, rinsed in distilled water 3X’s and then dehydrated in a graded series of ethanol as follows: 50 %, 70 %, 80 %, 95 %, 100 %, 100 % 10 min each step. Cells were placed into propylene oxide for 20 min, then into a 50:50 mixture of propylene oxide and Embed 812 resin medium overnight at room temperature. Samples were cured overnight in beem capsules at 60 degrees and then sectioned with a diamond knife on a Leica UC-7. Sections were cut at 80 nm and contrasted with uranyl acetate and lead citrate and imaged on a JEOL 100CX II Transmission Electron Microscope (Tokyo, Japan).

### Statistical analysis

At least three separate experiments were performed for each measurement unless otherwise indicated. All quantitative data were expressed as means with error bars representing 1 standard deviation (mean ± 1SD). Comparisons between two treatments were analyzed using a two-way student t-test with *P*-value of < 0.05 considered to be statistically significant. *p < 0.05, **p < 0.01, ***p < 0.001 unless otherwise indicated.

## Results

### Everolimus inhibits proliferation through induction of G1 arrest

**Fig. 1:**
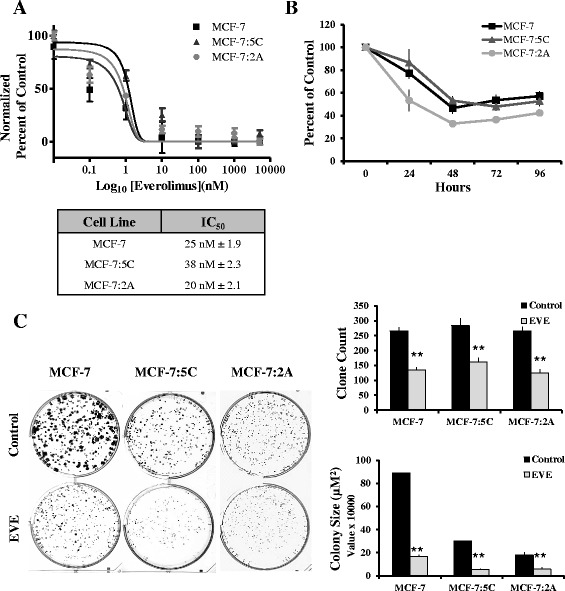
Everolimus inhibits the proliferation of AI-sensitive and AI-resistant breast cancer cells. **a** MCF-7, MCF-7:5C and MCF-7:2A cells were treated with 20 nM everolimus or vehicle (control) for 72 h. The percent of viable cells after everolimus treatment was determined by cell viability assay and compared to control. IC_50_ values for each of the cell lines were determined by nonlinear regression on normalized values. Values represent means of three experiments conducted in quadruplet. **b** MCF-7, MCF-7:5C and MCF-7:2A cells were seeded in 24-well plates and treated with 20 nM everolimus or vehicle after overnight acclimatization (Day 0). The percentage of viable cells was determined at 24, 48, 72 and 96 h post treatment by comparison to vehicle treated cells. **c** Cells were seeded in single cell suspension and allowed to proliferate for 9 days in the presence of 20nM everolimus or vehicle (*control*). The plates were photographed at 1X magnification (left panel) and the number of colonies and colony size were quantified using Image J (*right panel*). Bar graphs represent the data from three independent experiments in triplicate and values are mean ± SD. ** *p* < 0.01

We tested the anti-proliferative effect of everolimus in two AI-resistant breast cancer cell lines, MCF-7:5C and MCF-7:2A and their parental AI-sensitive cell line, MCF-7. We found that everolimus inhibited the proliferation of MCF-7:5C and MCF-7:2A cells with relatively equal efficiency compared to MCF-7 breast cancer cells (Fig. 1a, upper panel). The IC_50_ values for MCF-7, MCF-7:5C and MCF-7:2A cells were 25 nM, 38 nM and 20 nM, respectively, indicating only minor differences in sensitivity to everolimus between these cell lines (Fig. 1a, lower panel). Treatment with 20 nM everolimus achieved maximal inhibition of all three cell lines (Fig. 1a) as early as 24 h post-treatment (Fig. 1b). Additionally, the everolimus mediated inhibition of proliferation could be maintained with treatment every three days under clonogenic assay conditions (Fig. 1c). In contrast, everolimus had no effect on the proliferation of the immortalized normal breast epithelium cell line, MCF10A (Additional file 1: Figure S1a and b).

**Fig. 2:**
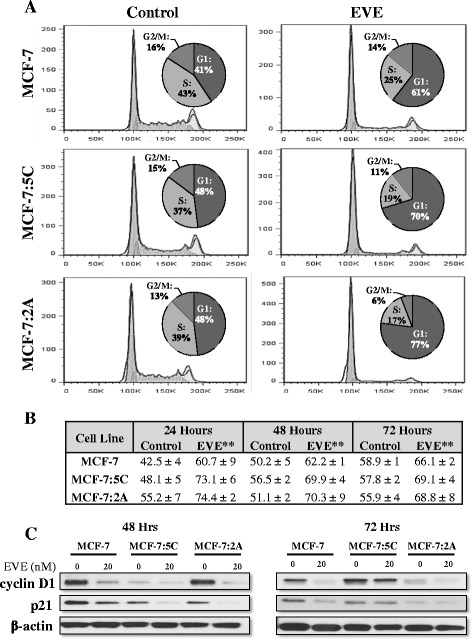
Everolimus induces G1 arrest. **a** MCF-7, MCF-7:5C and MCF-7:2A cells were treated for 24 h with 20 nM everolimus or vehicle and then harvested by trypsinization. Samples were fixed with methanol, stained with propidium iodide and analyzed by flow cytometry. The percent of cells in each phase of the cell cycle from a representative experiment are indicated in pie charts. **b** Samples from cells treated for 24, 48 and 72 h are summarized in the table. Values are means from three independent experiments analyzed in duplicate and are displayed as mean ± SD. ** *p* < 0.01. **c** Cyclin D1 and p21 expression in MCF-7, MCF-7:5C and MCF-7:2A cells following treatment with everolimus (20 nM) for 48 and 72 h

Cell cycle analysis of MCF-7, MCF-7:5C, and MCF-7:2A cells treated with everolimus indicated that the anti-proliferative effect of the drug was due to G1 arrest. The percentage of cells in G1 phase increased by at least 20 % in all three cells lines as early as 24 h after treatment (Fig. 2a) and this persisted through 72 h (Fig. 2b). Everolimus had no effect on the cycling of the normal breast epithelial cell line MCF10A (Additional file 1: Figure S1c). Additionally, we found that the expression of cyclin D1 and p21 were significantly reduced in MCF-7, MCF-7:5C and MCF-7:2A cells 48 and 72 h after treatment (Fig. 2c). This data indicates that everolimus is effective at inhibiting the proliferation of breast cancer cells due to marked induction of G1 arrest.

### Everolimus reduces the anchorage-independent growth

**Fig. 3:**
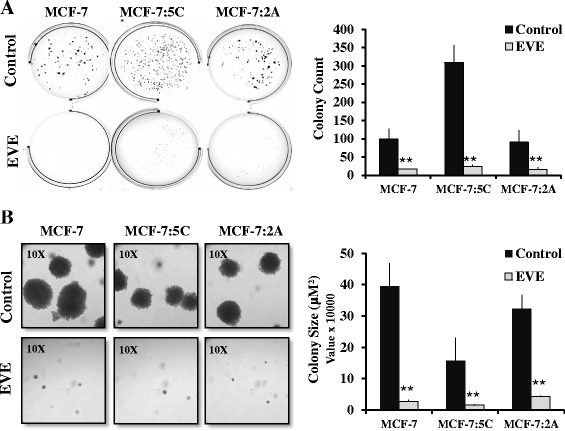
Everolimus reduces anchorage-independent growth. **a** MCF-7, MCF-7:5C and MCF-7:2A cells were seeded in single cell suspension within a layer of 0.5 % agarose gel. Cells were treated with 20 nM everolimus or vehicle every 48 h for 17 days. Images were taken at 1X magnification and the number of colonies quantified using Image J. **b** Images of the same wells were taken at 10X magnification and colony diameter in μm^2^ is shown. Bar graphs represent the data from three independent experiments in duplicate. The displayed values are mean ± SD. ** *p* < 0.01

The ability of cancer cells to grow in an anchorage-independent manner is a critical marker of tumor forming and metastatic potential. We compared the abilities of MCF-7, MCF-7:5C and MCF-7:2A cells to grow in an anchorage-independent manner using the soft-agar 3D colony formation assay. We found that MCF-7:5C cells produced three times more 3D colonies than the MCF-7 and MCF-7:2A cells. Additionally, 20 nM everolimus significantly reduced the number of 3D colonies in all three cell lines (Fig. 3a). The 3D colonies formed by the MCF-7:5C cells averaged 18 μM^2^, while those formed by the MCF-7 and MCF-7:2A cells averages 40 and 35 μM^2^ respectively. Upon microscopic inspection, we found that 20 nM everolimus dramatically reduced the size of 3D colonies in all three cell lines, with the most pronounced effect being on the MCF-7:5C cells (Fig. 3b). These data indicate that everolimus inhibits not just the proliferation of breast cancer cells, but also their tumor forming and metastatic potential.

### Effects of everolimus on the PI3K/Akt/mTOR pathway

**Fig. 4:**
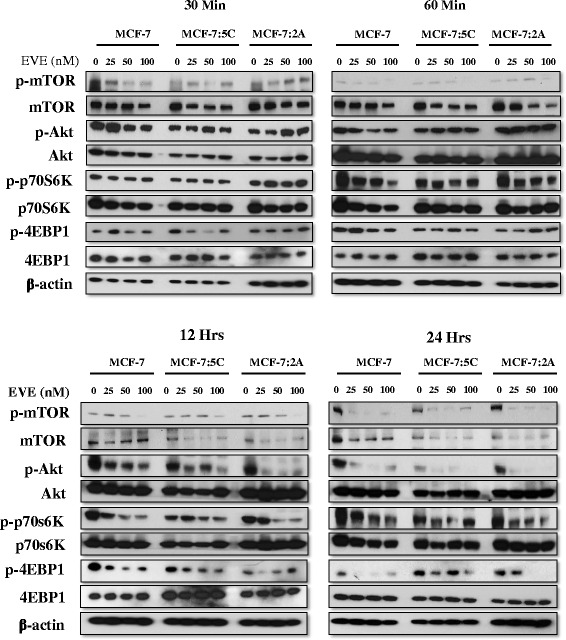
Everolimus targets the mTOR/Akt pathway. AI-sensitive MCF-7 and AI-resistant MCF-7:5C and MCF-7:2A cells were treated with 25, 50 and 100nM everolimus. Phosphorylation of mTOR, AKT, p70s6K, 4EBP1 and total protein levels are shown at 30mins, 60mins, 12 and 24 h. Images are representative of three independent experiments

We also examined the effect of everolimus treatment on the activation of the PI3K/Akt/mTOR pathway. We found that everolimus significantly inhibited mTOR phosphorylation as early as 30 min post treatment but not Akt, p70S6K and 4EBP1 (Fig. 4, upper panels). Everolimus inhibited the phosphorylation of downstream members of the PI3K/Akt/mTOR pathway at 12 h. This was most prominent at 24 h in MCF-7, MCF-7:5C and MCF-7:2A cells (Fig. 4, bottom panels). 25 nM everolimus was sufficient to inhibit PI3K/mTOR/Akt signaling at 12 and 24 h but higher doses were more effective at blocking phosphorylation. Notably, inhibition of p70S6K phosphorylation was observed by 60 min in the AI-sensitive MCF-7 cell line, especially with higher doses, but not in the AI-resistant MCF-7:5C and MCF-7:2A cells until 12 h post-treatment (Fig. 4). Reduction of phospho- mTOR, p70S6K and Akt were maintained by 20 nM everolimus through 48 and 72 h (Additional file 2: Figure S2). Everolimus successfully targets the Akt/mTOR pathway in AI-sensitive and AI-resistant breast cancer cells.

### Everolimus reduces estrogen receptor (ER) expression and transcriptional activity

**Fig. 5:**
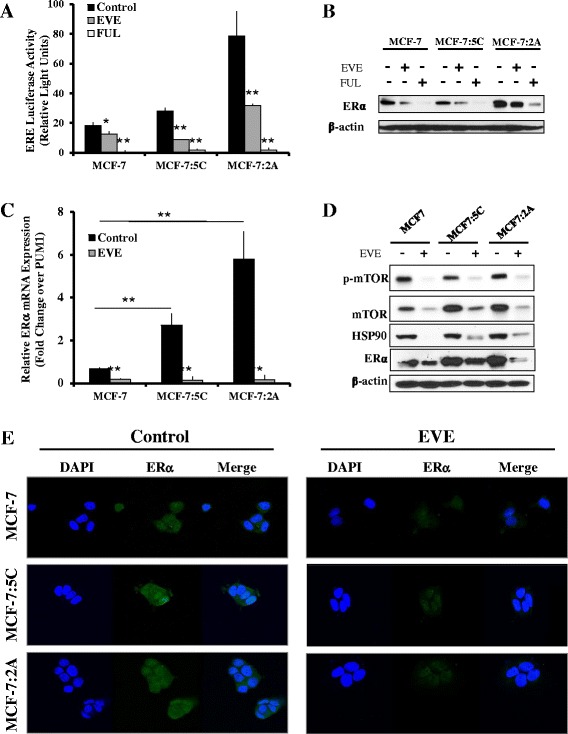
Everolimus treatment inhibits estrogen receptor α expression and activity. **a** ER transcriptional activity after 24 h treatment of MCF-7, MCF-7:5C and MCF-7:2A cells with 20 nM everolimus (EVE) and 1 μM fulvestrant (FUL) is demonstrated using an ERE luciferase reporter. **b** Corresponding expression of estrogen receptor α (ERα) after 48 h of treatment is shown by western blot. **c** RT-PCR demonstrates the impact of 20 nM everolimus on ERα mRNA production in all three cell lines after 24 h. **d** The effect of everolimus treatment on mTOR phosphorylation as well as HSP90 and ERα expression is shown. **e** Immunofluorescent confocal microscopy illustrates the impact of 20 nM everolimus on ERα expression and localization (green). Cell nuclei are stained with DAPI (blue). Bar graphs represent means from two independent experiments analyzed in quadruplet and are displayed as mean ± SD. ** *p* < 0.01

The activity of ER can be regulated by the PI3K/Akt/mTOR pathway and is critical to the survival and proliferation of AI-sensitive MCF-7 cells, as well as the AI-resistant MCF-7:5C and MCF-7:2A cell lines. The ligand-independent activity of ER maintains the growth and survival of AI-resistant breast cancer cells [35, 36]. We found that treatment with everolimus significantly reduced ER transcriptional activity and protein expression (Fig. 5a and b). This was compared to the action of the pure anti-estrogen fulvestrant (Fig. 5a and b). Everolimus also dramatically reduced ERα mRNA expression (Fig. 5c) in addition to protein expression of the ER chaperone, HSP90 (Fig. 5d). Downregulation of ER expression was confirmed by immunofluorescent confocal microscopy (Fig. 5e). Notably, there was higher ER transcriptional activity in AI-resistant MCF-7:5C and MCF-7:2A cells compared to parental MCF-7 cells, confirming estrogen-independent ER action in the resistant cells (Fig. 5a). Taken together, these data indicate that everolimus, and therefore PI3K/Akt/mTOR signaling, is capable of regulating ER expression and transcriptional activity in both wild-type MCF-7 cells and AI-resistant MCF-7:5C and MCF-7:2A cells.

### Everolimus reverses 4-OH tamoxifen resistance

**Fig. 6:**
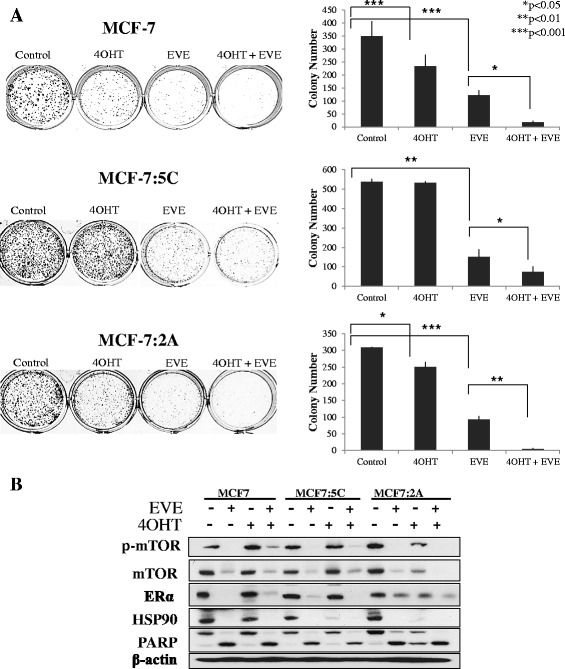
Everolimus enhances tamoxifen sensitivity. **a** MCF-7, MCF-7:5C and MCF-7:2A cells were seeded in single cell suspension and allowed to proliferate for 9 days in the presence of 20 nM everolimus, 1 μM 4-hydroxy tamoxifen (4OHT) or vehicle for 48 h. The plates were photographed and the number of colonies was quantified using Image J. Bar graphs represent the data from three independent experiments in triplicate and values are mean ± SD. *p < 0.05, **p < 0.01. *** *p* < 0.001. **b** Western blot indicates the effect of each treatment on mTOR phosphorylation and ERα, HSP90 expression and cleaved PARP

Due to earlier studies that have found synergy between tamoxifen and everolimus in endocrine-sensitive breast cancer cell models and patients, we investigated the efficacy of this combination in our MCF-7, MCF-7:5C and MCF-7:2A cells. We have previously shown that the AI-resistant MCF-7:5C cells are not responsive to 4OHT, whereas MCF-7:2A are partially sensitive to 4OHT [36]. In this study, 1 μM 4OHT significantly inhibited the proliferation of MCF-7 and MCF-7:2A cells, reducing the number of 2D colonies by 20 % and 10 % respectively, but had no effect on MCF-7:5C cells (Fig. 6a). Treatment with 20 nM everolimus for 9 days significantly reduced the proliferation of all three cell lines, reducing colony numbers by ~ 60 % (Fig. 6a). Co-treatment of MCF-7, MCF-7:5C and MCF-7:2A cells with 1 μM 4OHT and 20 nM everolimus reduced colony formation in MCF-7 and MCF-7:2A cells by ~ 95 % and also had added benefit in the 4OHT-resistant MCF-7:5C cells, bringing the anti-proliferative effect from 60 % to 76 %. Synergy between 4OHT and everolimus treatment was present despite reductions in ERα expression in all three cell lines (Fig. 6b). This data supports clinical observations that the combination of tamoxifen with everolimus has therapeutic benefit in patients with ER+ breast cancer and can re-sensitize AI-resistant breast cancer to endocrine therapy.

### Everolimus induces autophagy in breast cancer cells, which mediates insensitivity

**Fig. 7:**
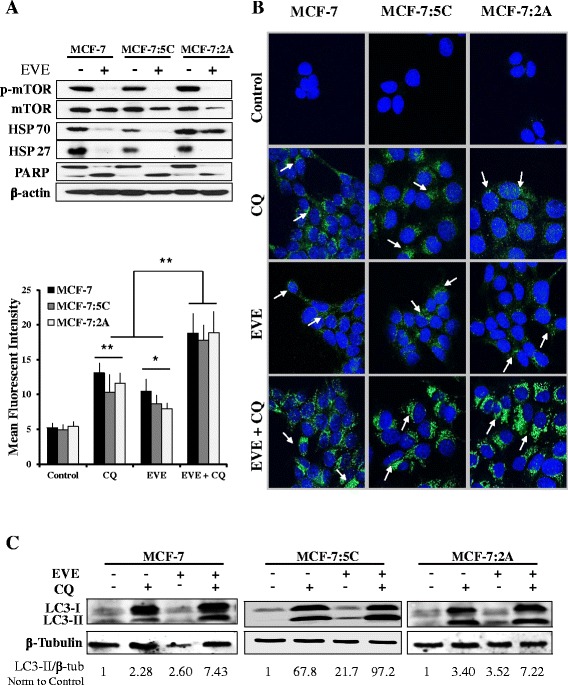
Everolimus modifies autophagic cell markers. **a** Inhibition of mTOR phosphorylation, downregulation of HSP70 and HSP27 as well as induction of PARP cleavage by 20 nM everolimus are shown by western blot. **b** MCF-7, MCF-7:5C and MCF-7:2A cells were treated with vehicle, 20 nM everolimus, 50 μM chloroquine (CQ) or both for 24 h. LC3B is shown by immunofluorescent confocal microscopy. Punctate LC3B granules (green) are indicated with white arrows. Cell nuclei are stained with DAPI (blue). Mean fluorescent intensity was determined using Image J. **c** Cells were treated with vehicle, 20 nM everolimus, 50 μM chloroquine (CQ) or both for 48 h and LC3-I and -II assessed by western blot. The ratio of LC3-II/β-Tubulin normalized to control was determined by densitometry with Image J and is shown below the blots

**Fig. 8:**
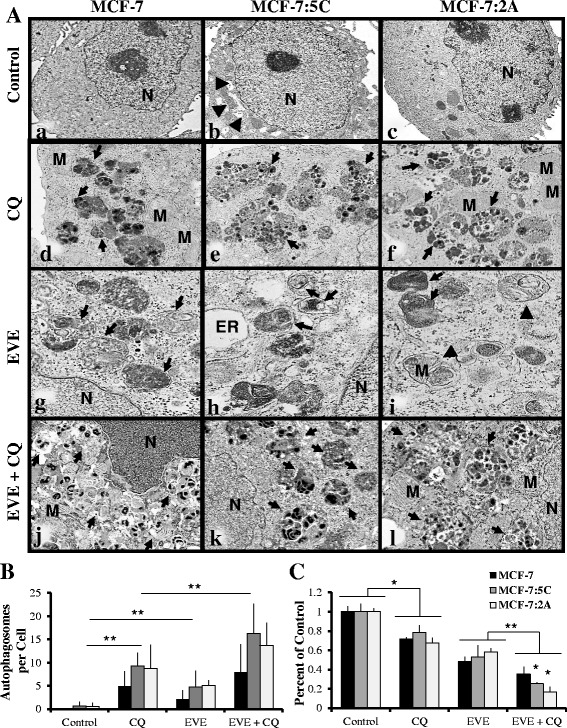
Everolimus treatment induces autophagy, which mediates insensitivity. **a** MCF-7, MCF-7:5C and MCF-7:2A cells were treated with vehicle (a-c), 50 μM chloroquine (CQ) (d-f), 20 nM everolimus (EVE) (g-i) or both EVE and CQ (j-l) for 48 h and imaged using electron microscopy. Arrows indicate double membrane bound autophagosomes and arrow heads indicate pleomorphic mitochondria and dilated endoplasmic reticulum, which could also be seen in untreated MCF-7:5C cells. Images a-c are shown at 2000 X magnification while *d*-*f*, *g*-*i* and *j*-*l* are shown at 5000X, 8000X and 6000X respectively. M, mitochondria; N, nucleus; ER, endoplasmic reticulum. **b** Autophagosomes in a minimum of 10 random cells were recorded using de-identified samples. Values are average autophagosomes per cell and represent three trials from two separate experiments. **c** Cells were seeded in 24-well plates and treated with vehicle, 20 nM everolimus (EVE), 50 μM chloroquine or both for 7 days. The percentage of viable cells is shown as compared to vehicle treated cells. Bar graphs represent the data from three independent experiments in duplicate and values are mean ± SD. * *p* < 0.05, ** *p* < 0.01

We investigated whether everolimus treatment induced autophagy in MCF-7, MCF-7:5C and MCF-7:2A cells. We found that everolimus reduced the levels of HSP70 and HSP27 in all three cell lines (Fig. 7a). Interestingly, everolimus also induced PARP cleavage (Fig. 7a); however, this was not associated with apoptosis by annexin v/PI staining (data not shown). Chloroquine was used as an autophagic flux inhibitor, and basal autophagy was assessed with and without everolimus treatment. Both immunofluorescent microscopy and western blot (Fig. 7b and 7c), indicate that everolimus markedly enhanced LC3-II above basal level, respectively. A lysosomal protease inhibitor cocktail (100 μM leupeptin, 10 μg/mL pepstatin A and 10 μg/mL e-64d for 24 h) was also used to inhibit autophagic flux but results were not as robust as with 50 μM chloroquine (data not shown). As further indication of everolimus’ ability to induce autophagy, the number of autophagosomes identified by electron microscopy in all three cell lines was also increased (Fig. 8a and 8b). Combined inhibition of autophagy with 50 μM CQ significantly improved the efficacy of everolimus treatment on cell proliferation, indicating that autophagy is a method of everolimus insensitivity in MCF-7 cell lines (Fig. 8c). Under normal conditions, the MCF-7:5C cell line displayed dilated endoplasmic reticulum, and both AI-resistant cell lines had pleomorphic mitochondria, indicating that aberrant metabolism is likely part of the phenotype of these AI-resistant breast cancer models (Fig. 8a). These data suggest that MCF-7, MCF-7:5C and MCF-7:2A cells use autophagy as a method of everolimus resistance and that this response may be inhibited to improve the efficacy of everolimus treatment in breast cancer.

## Discussion

This study was conducted to provide mechanistic insights into the anti-proliferative effects of everolimus in AI-resistant MCF-7:5C and MCF-7:2A breast cancer cells, and AI-sensitive MCF-7 cells. We found that everolimus was equally effective against all three breast cancer cell lines. The anti-proliferative mechanisms included downregulation of ER expression and transcriptional activity, possibly through the suppression of HSP90. We also demonstrated that everolimus enhanced 4OHT sensitivity in all three cell lines. Everolimus treatment significantly induced autophagy, which was associated with downregulation of HSP70 and HSP27 expression. Additionally, we confirmed that everolimus inhibits the activation of the PI3K/Akt/mTOR pathway, resulting in the downregulation of cyclin D1 and p21 expression, which induced G1 arrest.

MCF-7 cells and their derivatives are more resistant to everolimus as compared to other luminal A breast cancer cell lines [37, 38]. Our study is consistent with this observation, as total inhibition of the MCF-7, MCF-7:5C and MCF-7:2A cells did not exceed 60 %, making them suitable to model a patient population that is not highly sensitive to everolimus. Additionally, our IC_50_ values were consistent with the frequent use of 20 nM everolimus when studying MCF-7 cell lines [17, 39, 40]. The enhanced insensitivity of the MCF-7:5C cells may be due to increased expression of c-myc, which is thought to confer some resistance in ER+ breast cancer [41, 42]. The slightly enhanced sensitivity of the MCF-7:2A cells to everolimus may be related to comparatively lower levels of PTEN [42]. It should be noted that the MCF-7 and MCF-7:2A cells are progesterone receptor-positive (PR+), while the MCF-7:5C cells are PR-negative (PR-) and they overexpresses interferon stimulated genes [43]. These differences do not seem to mediate any variances in everolimus sensitivity, suggesting that the MCF-7 background of these cell lines is the most prominent determinant of response.

Elevated ER expression and signaling has been observed in both endocrine resistance cells and endocrine resistant tumors [2, 3, 44, 45]. The ability of everolimus to reduce ER transcriptional activity and phospho-ER (p-ER) expression in MCF-7/LTED cells has been previously reported but was not assessed in wild type MCF-7 cells [15]. In our study, we found that the inhibition of ER transcriptional activity was due to profound downregulation of total ER expression in all cell models. MCF-7:5C and MCF-7:2A cells are selected clones maintained in estrogen-free media that have retained ER transcriptional activity by upregulation of ER expression and ligand-independent ER signaling. In contrast, the MCF-7/LTED cells are a mixed population of cells that have developed hypersensitivity to estrogen [46]. Additionally, the studies by Martin and colleagues were conducted after acute insulin deprivation, which probably contributed to the enhanced sensitivity to everolimus and to the impact on both p-ER and p-Akt expression in their study. The clinical efficacy of everolimus as a solo agent was thought to be limited by a compensatory increase in Akt phosphorylation through mTORC2 [17]. We did not observe this reflexive increase in Akt phosphorylation in our cells lines. Our results suggest that everolimus as a single agent has the ability to function in a manner similar to combination therapy by inhibiting both growth factor and ER signaling simultaneously in some systems. Downregulation of total ER expression by everolimus has not, to our knowledge, been previously reported.

The downregulation of ER expression was likely due to reduced expression of HSP90, a well-known ER chaperone. HSP expression in general is controlled by the PI3K/Akt/mTOR pathway through phosphorylation of the transcription factor HSF1 and is consistent with the loss of HSP90, HSP27 and HSP70 expression observed in this study [47]. Everolimus induced loss of HSP90 mRNA expression has been observed in other cancers [19] and a loss of HSP90 expression has been linked to autophagic cell death [48]. Two HSP90 inhibitors, NVP-AUY922 and STA-9090, are currently in clinical trials for the treatment of breast cancer [49, 50]. HSP90 inhibitor therapy has been limited by reflexive increase in HSP signaling, especially enhanced HSP27 expression [51, 52]. Here, we report that everolimus inhibits HSP27 in our cell lines, and so may potentiate HSP90 inhibitor treatment. These results suggest that everolimus may be combined with HSP90 inhibitors or drugs that target HSP90 clients for the treatment of AI-resistant breast cancer.

AI-resistant tumors are known to retain dependence on ER signaling for growth and survival. Given that everolimus reduces ER expression, our observation that it also enhances 4OHT sensitivity during long-term co-treatment is very interesting and warrants further investigation. It should be noted, however, that everolimus has previously been shown to inhibit ER phosphorylation on serine 167 despite documented synergy between everolimus and tamoxifen in AI-resistant models and patients [10, 15, 17]. It is likely that these two drugs exhibit synergy by targeting ER signaling through separate but complementary mechanisms. Our results indicate that everolimus reduces ER expression through inhibition of ER mRNA transcription, while tamoxifen targets the ER protein. Combining everolimus and tamoxifen ensures that ER signaling is inhibited continuously over time in all cells within a heterogeneous breast tumor. The improved efficacy of everolimus in combination with 4OHT in our study is consistent with results from the TAMRAD clinical trial, [10]. The data from our study suggest that everolimus could benefit AI-resistant patients with ligand-independent ER activity by targeting ER expression and signaling.

We have demonstrated that everolimus dramatically induces autophagy, and is associated with significant downregulation of HSP90, HSP70 and HSP27. Loss of HSP70 and HSP27 is associated with starvation-induced autophagy and rapamycin treatment, both of which target the PI3K/Akt/mTOR pathway [26, 30]. Autophagy is a mechanism of drug insensitivity in cancer because it allows tumors to recycle cellular components and survive treatment [21, 22]. Inhibition of autophagy significantly improved the anti-proliferative effects of everolimus in MCF-7, MCF-7:5C and MCF-7:2A cells. This is consistent with a previous study reporting enhanced inhibition of MCF-7 cell proliferation when combining chloroquine and everolimus [53]. Everolimus and other PI3K/Akt/mTOR inhibitors are known to induce autophagy in both solid and blood tumors [19, 20]; however, to our knowledge, this phenomenon has not been reported in breast cancer cells. We conclude that the induction of autophagy is likely a mechanism of everolimus insensitivity in MCF-7 [37, 38], MCF-7:5C and MCF-7:2A cells.

Although everolimus treatment induced PARP cleavage in all three cell lines, we did not observe apoptotic cell death normally associated with PARP cleavage. PARP cleavage is thought to mediate autophagy rather than apoptosis in response to certain stimuli [54]. Since HSP70 is known to stabilize PARP [55], loss of HSP70-mediated stability offers an explanation for the everolimus induced PARP cleavage seen in this study. While there have been reports of autophagic and apoptotic cell death in leukemia [19] and nasopharyngeal carcinoma cells [56], everolimus is not known to induce cell death in breast cancer cells on its own [19, 56]. To our knowledge, everolimus induced cell death in breast cancer has only been observed in aromatase expressing MCF-7/Aro cells when combined with letrozole [7]. We have shown that induction of autophagy limits the anti-proliferative response of everolimus treatment, hence a combination of everolimus with the autophagy inhibitors chloroquine or hydroxychloroquine, which are currently in clinical trials as part of combination therapy [57], may be beneficial in the treatment of AI-resistant breast cancer.

## Conclusions

Overall, this study demonstrated that everolimus inhibits the proliferation of AI-resistant breast cancer cells through down regulation of ER expression and also that induction of autophagy is a method of everolimus insensitivity. We found that everolimus had similar effect on the proliferation of both our AI-sensitive (MCF-7) and AI-resistant (MCF-7:5C and MCF-7:2A) models, suggesting that the MCF-7 background of these cell lines overrides any other differences that might impact everolimus sensitivity. The inhibition of proliferation was seen regardless of PR status, PTEN expression, type 1interferon signaling, and 4OHT sensitivity, supporting a conclusion that everolimus holds promise as part of combination therapy for a wide variety of AI-resistant patients, for whom AI treatment is not an option.

## Abbreviations

AI, aromatase inhibitor; CQ, chloroquine; EGF, epidermal growth factor; ER, estrogen receptor; EVE, everolimus; FUL, fulvestrant; HER2, human epidermal growth factor receptor 2; HIF-1α, hypoxia inducible factor 1 alpha subunit; HSP27, Heat shock protein 27; HSP70, heat shock protein 70; HSP90, heat shock protein 90; IC_50_, drug concentration that provides 50 % maximal growth inhibition; LC3B, Microtubule associated light chain 3; mTOR, mammalian target of rapamycin; PARP, Poly ADP ribose polymerase; PI3K, phosphoinositide 3-kinase; PR, Progesterone receptor; PTEN, phosphatase and tensin homolog; P21, cyclin dependent kinas inhibitor 1; p70S6K, p70 S6 ribosomal protein kinase; TSC, tuberous sclerosis complex; 4OHT, 4- hydroxytamoxifenv; 4EBP1, Eukaryotic translation initiation factor 4E-bidning proteinv.

## Additional files

Additional file 1: Figure S1. Everolimus does not impact the proliferation or cycling of normal breast cells. (**a**) MCF10A cells were seeded in 24-well plates and treated with a range of everolimus doses in triplicate. The percentage of viable cells was determined after 72 h of everolimus treatment. (**b**) MCF10A cells were seeded in 6-well plates in single cell suspension and treated with 20 nM everolimus for 9 days. The number and size of clones was quantified and represents means from two independent experiments conducted in triplicate. (**c**) After 24, 48 and 72 h of 20 nM everolimus treatment in 6-well plates, MCF10A cells were subjected to cell cycle analysis. The percent of cells in G1 phase is highlighted. (PPT 507 kb) Additional file 2: Figure S2. Everolimus targets the phosphorylation of the PI3K/mTOR/Akt pathway at 48 and 72 h. MCF-7, MCF-7:5C and MCF-7:2A cells were seeded in 6-well plates and treated with 25, 50 or 100 nM everolimus or vehicle. Cells were harvested at 48 and 72 h and protein expression analyzed by western blot. Image represents three independent experiments. (PPT 674 kb)
