# Supplementary material for: Everolimus downregulates estrogen receptor and induces autophagy in aromatase inhibitor-resistant breast cancer cells
Source: BMC Cancer. 2016 Jul 16;16:487. doi: 10.1186/s12885-016-2490-z (PMC4947349; doi:10.1186/s12885-016-2490-z)
Supplement: Additional file 1: Figure S1. — Everolimus does not impact the proliferation or cycling of normal breast cells. (a) MCF10A cells were seeded in 24-well plates and treated with a range of everolimus doses in triplicate. The percentage of viable cells was determined after 72 h of everolimus treatment. (b) MCF10A cells were seeded in 6-well plates in single cell suspension and treated with 20 nM everolimus for 9 days. The number and size of clones was quantified and represents means from two independent experiments conducted in triplicate. (c) After 24, 48 and 72 h of 20 nM everolimus treatment in 6-well plates, MCF10A cells were subjected to cell cycle analysis. The percent of cells in G1 phase is highlighted. (PPT 507 kb) [file 12885_2016_2490_MOESM1_ESM.ppt]

## Slide 1
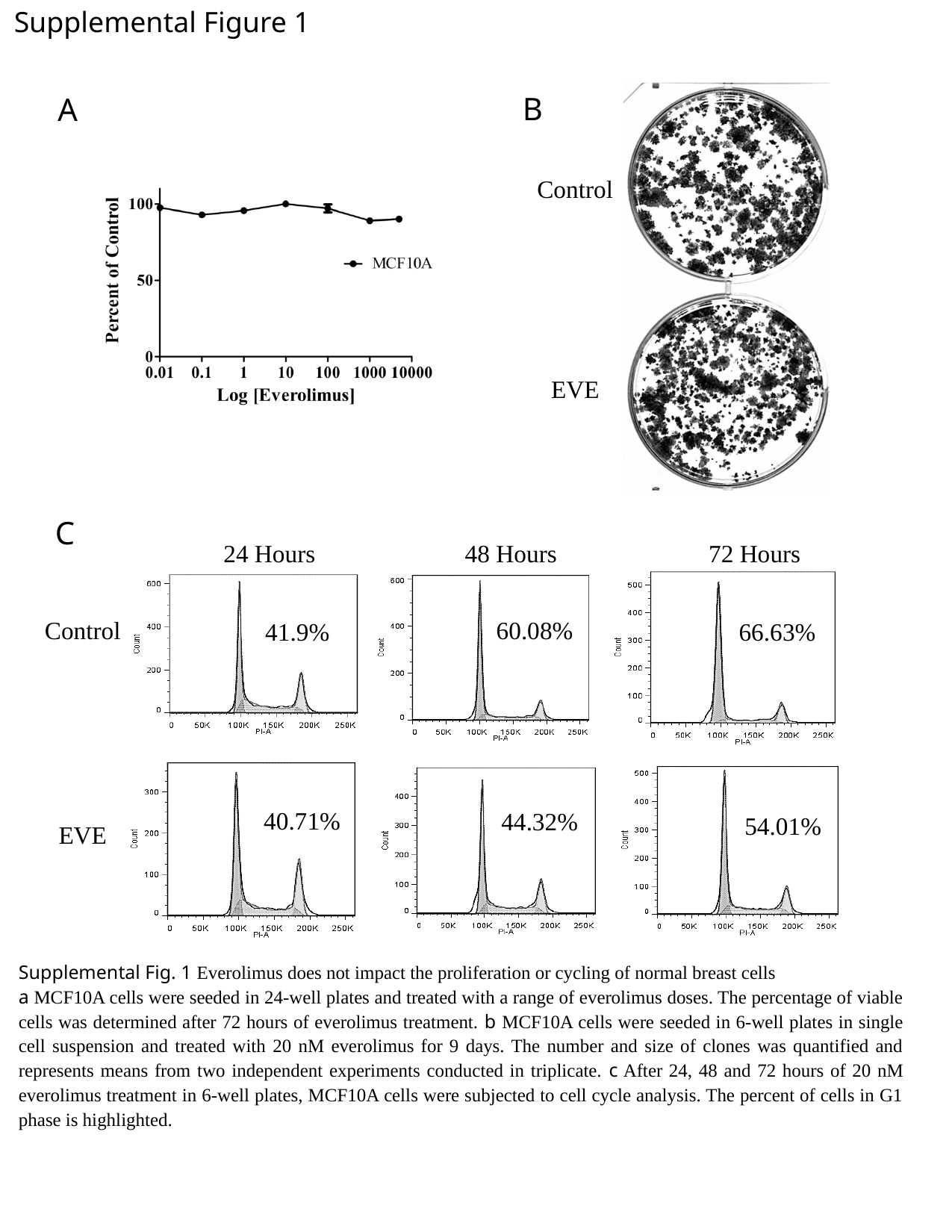

Supplemental Figure 1
Control
EVE
B
A
C
24 Hours
48 Hours
72 Hours
Control
60.08%
41.9%
66.63%
44.32%
54.01%
EVE
40.71%
Supplemental Fig. 1 Everolimus does not impact the proliferation or cycling of normal breast cells
a MCF10A cells were seeded in 24-well plates and treated with a range of everolimus doses. The percentage of viable cells was determined after 72 hours of everolimus treatment. b MCF10A cells were seeded in 6-well plates in single cell suspension and treated with 20 nM everolimus for 9 days. The number and size of clones was quantified and represents means from two independent experiments conducted in triplicate. c After 24, 48 and 72 hours of 20 nM everolimus treatment in 6-well plates, MCF10A cells were subjected to cell cycle analysis. The percent of cells in G1 phase is highlighted.
