# Supplementary material for: Everolimus downregulates estrogen receptor and induces autophagy in aromatase inhibitor-resistant breast cancer cells
Source: BMC Cancer. 2016 Jul 16;16:487. doi: 10.1186/s12885-016-2490-z (PMC4947349; doi:10.1186/s12885-016-2490-z)
Supplement: Additional file 2: Figure S2. — Everolimus targets the phosphorylation of the PI3K/mTOR/Akt pathway at 48 and 72 h. MCF-7, MCF-7:5C and MCF-7:2A cells were seeded in 6-well plates and treated with 25, 50 or 100 nM everolimus or vehicle. Cells were harvested at 48 and 72 h and protein expression analyzed by western blot. Image represents three independent experiments. (PPT 674 kb) [file 12885_2016_2490_MOESM2_ESM.ppt]

## Slide 1
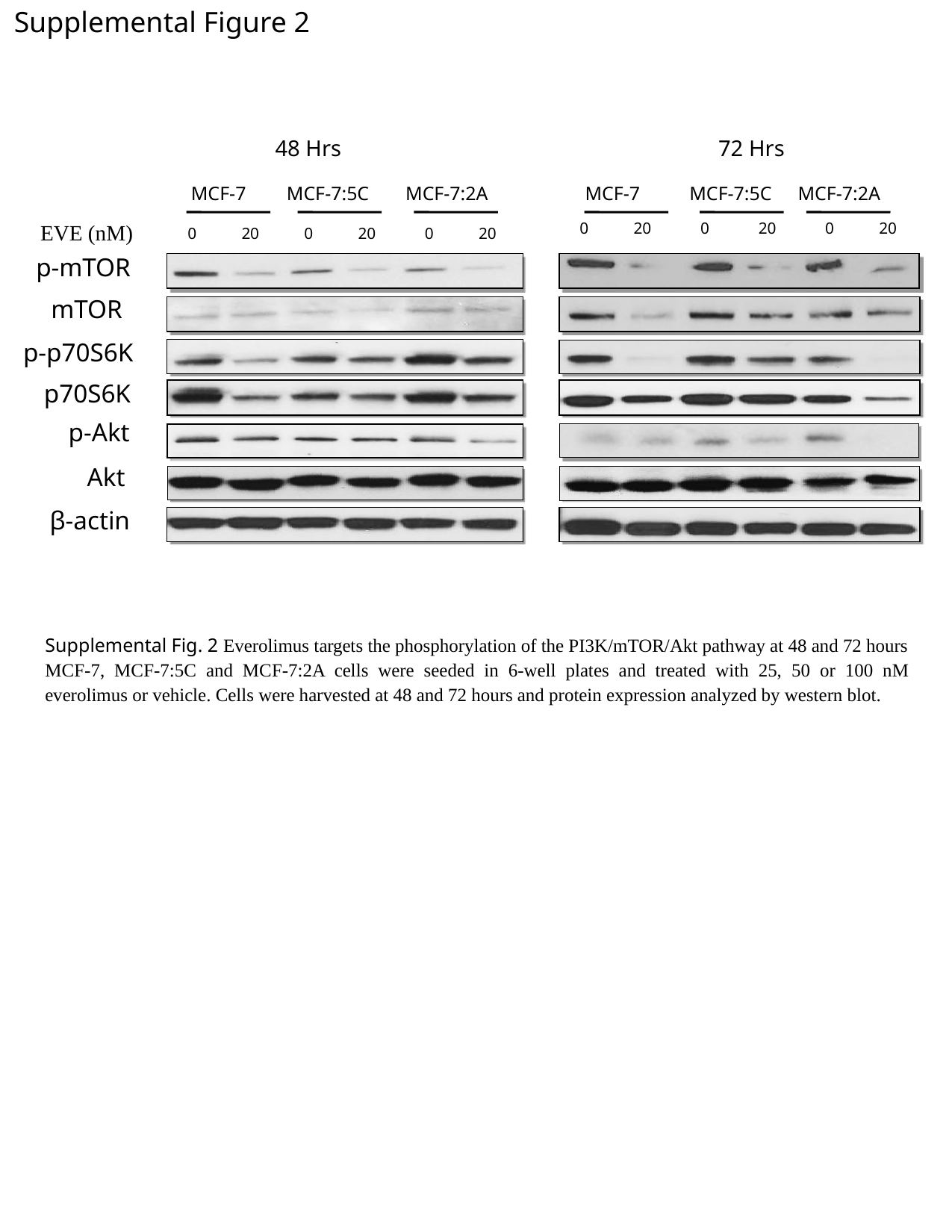

Supplemental Figure 2
48 Hrs
72 Hrs
MCF-7
MCF-7:5C
MCF-7:2A
MCF-7
MCF-7:5C
MCF-7:2A
EVE (nM)
0 20 0 20 0 20
p-mTOR
mTOR
p-p70S6K
p70S6K
p-Akt
Akt
β-actin
0 20 0 20 0 20
Supplemental Fig. 2 Everolimus targets the phosphorylation of the PI3K/mTOR/Akt pathway at 48 and 72 hours
MCF-7, MCF-7:5C and MCF-7:2A cells were seeded in 6-well plates and treated with 25, 50 or 100 nM everolimus or vehicle. Cells were harvested at 48 and 72 hours and protein expression analyzed by western blot.
